# Locus-specific Retention Predictor (LsRP): A Peptide Retention Time Predictor Developed for Precision Proteomics

**DOI:** 10.1038/srep43959

**Published:** 2017-03-17

**Authors:** Wenyuan Lu, Xiaohui Liu, Shanshan Liu, Weiqian Cao, Yang Zhang, Pengyuan Yang

**Affiliations:** 1 Institutes of Biomedical Sciences and Department of Systems Biology for Medicine, School of Basic Medical, Fudan University, Shanghai, 200032, P. R. China; 2 Department of Chemistry, Fudan University, Shanghai, 200433, P. R. China

## Abstract

The precision prediction of peptide retention time (RT) plays an increasingly important role in liquid chromatography–tandem mass spectrometry (LC–MS/MS) based proteomics. Owing to the high reproducibility of liquid chromatography, RT prediction provides promising information for both identification and quantification experiment design. In this work, we present a Locus-specific Retention Predictor (LsRP) for precise prediction of peptide RT, which is based on amino acid locus information and Support Vector Regression (SVR) algorithm. Corresponding to amino acid locus, each peptide sequence was converted to a featured locus vector consisting of zeros and ones. With locus vector information from LC-MS/MS data sets, an SVR computational process was trained and evaluated. LsRP finally provided a prediction correlation coefficient of 0.95~0.99. We compared our method with two common predictors. Results showed that LsRP outperforms these methods and tracked up to 30% extra peptides in an extraction RT window of 2 min. A new strategy by combining LsRP and calibration peptide approach was then proposed, which open up new opportunities for precision proteomics.

Shotgun technique based on liquid chromatography (LC) and electrospray ionization-mass spectrometry (ESI-MS) is presently considered as a powerful and popular approach in the separation and identification of complex protein mixtures^1^,^2^. Widely applied MS-based workflows consists of the same main steps^3^,^4^: Sequence-specific enzymes are used to cleave proteins into peptides, which are suitable for separation on a LC column. Then separated peptides are ionized and subjected to MS analysis. Spectra from tandem MS are dealt with proteomics software package to identify and quantify the peptides, such as Mascot and Maxquant^5^. However, the complexity of proteome makes accurate peptide/protein identification more challenging^6^. It is proposed that additional information besides the fragmentation spectra should be utilized. One example of such additional information is peptide RT^7^,^8^.

LC-based separation reduces the number of peptides simultaneously injected into MS and increases the number of peptides selected for fragmentation^9^. In spite of small shifts between runs, each peptide elutes almost at the same time under certain experimental condition. In other words, RT is one of the important properties for peptides^10^. Peptide retention prediction has recently gained lots of attentions in selected reaction monitoring (SRM)^11^,^12^,^13^ and data-independent-acquisition (DIA) workflows, such as SWATH^14^,^15^. To truly increase the coverage of a proteome and to achieve better SRM or SWATH experiment design, it would be required to involve precision RT prediction into present methods^16^,^17^,^18^.

For a long time, RT was predicted by a linear combination of the counts of a peptide’s amino acids weighted by a hydrophobicity value^19^. It is typically thought that the more hydrophobic the peptide is, the longer the retention time. However, hydrophobicity is still an ill-defined property^20^. Improving peptide RT prediction in RPLC requires an understanding of the various factors affecting RTs. And recently, there have been several studies on the RT prediction for reversed-phase liquid chromatography (RPLC). Two types of peptide properties are chosen as factors to predict RTs. One factor is based on quantitative structure-retention relationships (QSRR), and the other is based on peptide sequences.

QSRR features that describe molecular structure information are usually calculated with specialized molecular modeling software. B̧aczek *et al*. adopted Partial Least Squares (PLS) for regression, which is subsequently used to predict RT^21^. Afterwards, machine learning methods were applied. Tian *et al*. employed Support Vector Regression (SVR) to deal with the calculated molecular descriptors from QSRR and reported a satisfactory model performance^22^. Shinoda *et al*. and Petritis *et al*. respectively employed Artificial Neural Network (ANN) for development of QSRR data^23^,^24^. Lately, B̧aczek *et al*. compared the prediction accuracies of three mentioned machine learning methods PLS, SVR and ANN^25^. It is reported that all the models exhibit high predictive power, but SVR has shown to be superior with the lowest minimizing Root Mean Square Error of Prediction (RMSEP) for both the testing and validation set. All QSRR methods take long time to calculate for prediction^3^.

However, compared with QSRR features, features derived from the peptide’s sequence are acquired more conveniently. There are two well-trained predictors based on peptide sequence widely used in proteome analysis. Krokhin *et al*. developed Sequence Specific Retention Calculator (SSRCalc) that predicts RT with a linear regression of the Hydrophobicity Index (HI). HI was calculated by amino acid composition information such as peptide length, isoelectric point, and nearest-neighbor effects of charged peptides^26^,^27^,^28^,^29^. Käll *et al*. developed ELUDE that derives a RT index for the condition at hand making it fully portable to new chromatographic conditions^17^. It is reported that SSRCalc and ELUDE have been the most popular retention time predictors^3^. In addition to the mentioned packaged predictors, several machine learning methods were also introduced to model training with peptide sequence information. Petritis *et al*. applied ANN for prediction, using the numbers of each kind of amino acid in peptides as the input nodes^30^. Then Petritis firstly translated the peptide sequence information into locus vectors consisting of zeros and ones, which were used for RT prediction, together with peptide length and hydrophobic moment^31^. Petritis pointed out prediction based on amino acid locus was more accurate than that based solely on amino acid composition. However, large numbers of training peptides were needed for ANN algorithm. Pfeifer *et al*. considered regions (consecutive sequence positions) where the amino acid occurs as input features, instead of every amino acid at every position^32^,^33^,^34^. Using SVR in combination with a kernel function, Pfeifer provided a squared correlation coefficient of 0.95~0.96 with a dramatically reduced training set of only hundreds peptides, while Petritis offered a squared correlation coefficient of 0.967 with ANN algorithm using 344,611 training peptides. As both Petritis and Pfeifer mentioned, features used in ELUDE and SSRC, such as composition, sequence length, hydrophobic regions, can be fully reflected in locus vectors of amino acid sequence. In addition, there is a one-to-one correspondence between a locus vector and a peptide sequence for all peptides (≤25 length) in this work, which is different from previous predictors like SSRCalc and ELUDE.

As for the training algorithm, the advantages and disadvantages of ANN and SVR were valued. ANN is considered as a computational “black box” that based on empirical risk minimization^35^. Thus, ANN is easy to get into the local optimum and needs large training sets. On the contrary, SVR is derived from the strict mathematic concept and theory. SVR algorithm based on structural risk minimization can guarantee global optimal and good generalization ability^36^.

Finally, we improved and simplified the method proposed by Petritis of translating the whole amino acid locus information into locus vectors consisting of zeros and ones, which is believed to reflect almost all peptide features. And we expected that SVR algorithm can help us train prediction models faster and more accurately. In this work, we developed LsRP combining locus vectors and SVR algorithm, for peptide RT prediction. We used RTs of training peptides to build a model, and then evaluated this model with test set. With LsRP, we can predict RTs more accurately than other existing predictors. We further applied LsRP to the investigation of prediction under different chromatographic conditions and successfully acquired good correlation coefficients with calibration peptides.

## Results and Discussion

### Locus Recognition and SVR Training

**Figure 1:**
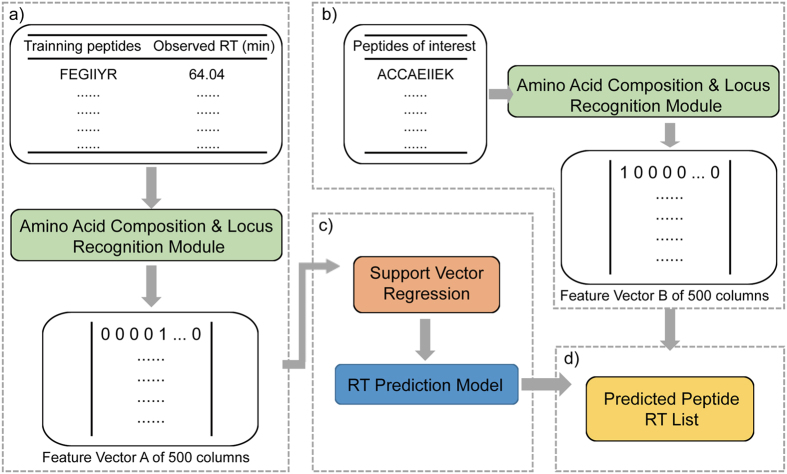
The LsRP workflow. (**a**) amino acid composition and locus recognition for training peptides, (**b**) amino acid composition recognition for interested peptides, (**c**) model construction via SVR method, (**d**) retention time prediction and export.

The RT predictor is based on the amino acid composition and locus recognition module as well as the core SVR algorithm (Fig. 1). To record amino acid order and locus in peptides, a series of vectors were designed as the idea of translating amino acid order into vectors proposed by Petritis (Supplementary Table S1). Each amino acid residue can be coded as a 20-dimensional binary vector consisting of 19 zero values and 1 one value that corresponds to the amino acid residue occupying that position. A theoretical digested peptide list for human proteins was then generated, which showed about 93% of peptides are shorter than 25 residues. In addition, it is also reported that it is hard to observe and identify peptides longer than 25 residues in ESI-MS^37^. Removing peptides longer than 25 residues lead to a final data set of about 97~98% identified peptides for our data sets (Table 1). In this work, length and hydrophobic moment were not employed for model training, because they are not independent variables and can be calculated from the locus vectors of peptide sequences. Petritis has also proved that the incorporation of the length and hydrophobic moment in the model is not as important as the incorporation of the peptide sequence^31^. Thus, in the following Amino Acid Composition and Locus Recognition Module, both training peptides and test peptides (≤25 length) were translated to locus vectors of 500 columns (20 amino acids × 25 length) filled with zeros and ones.

SVR is a machine learning method for linear and non-linear regression based on Support Vector Machines (SVM) invented by Vapnik^35^,^36^,^38^. Lin developed a series of optimized software named libsvm to apply SVM under programming language environments^39^. In this work, a function was introduced to approximate the high-dimensional data:


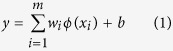


where 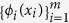 represents the features of input variables which are the corresponding vectors of 500 columns in this work, while 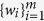 and b are coefficients. And a radial basis kernel function was employed to perform the non-linear mapping:


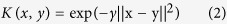


where *γ* is the radial basis function radius. After kernel transformation, the new feature space permits the data linearly distinguishable by hyperplanes. Then regularized risk function is defined as:


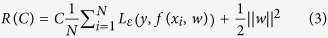


where the part before plus sign is the empirical error and the other is a measure of function flatness. The error is measured by the ε-insensitive loss function 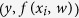 as following:


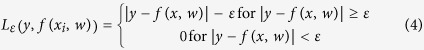


where the parameter *ε* is referred to as the tube size, which is the approximation accuracy placed on the training data points. Meanwhile, the punishment parameter C is a regularized constant responsible for determining the trade-off between the empirical error and the model complexity^25^. The purpose of SVR is to train a function that predicts all data within a given *ε* deviation from the actual values. And three parameters: *C, γ* and *ε*, can be figured out by a ranged exhaustive method to complete the regression.

### Performance of LsRP

**Figure 2:**
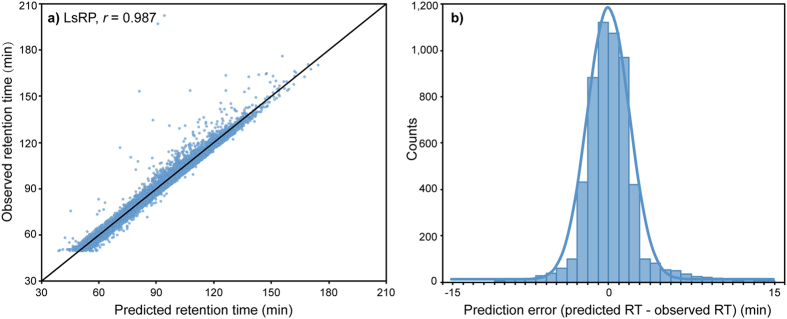
Prediction performance of LsRP for Hela 210 min data set. (**a**) Correlation between predicted and observed RTs, (**b**) Distribution of prediction error.

The performance of our RT predictor was evaluated by applying it to different data sets. The Hela 210 min data set was randomly divided into two equally sized group. One group was selected as training set and the other as test set. After model training, test peptides were processed by LsRP. Figure 2 shows the final prediction performance of the Hela 210 min data set. The predicted and observed RTs of the test peptides were plotted in Fig. 2a. The Pearson’s correlation of LsRP between predicted and observed RTs for the 5618 test peptides was *r* = 0.987. In addition, two common predictors (i.e. SSRCalc and ELUDE) on the web server were used to predict peptide RTs as comparisons. Supplementary Figure S1 shows that the Pearson’s correlations of SSRCalc and ELUDE are 0.966 and 0.959. Furthermore, we also generated histograms displaying the deviation between predicted and observed RTs of LsRP (Fig. 2b), SSRCalc and ELUDE (Supplementary Figure S2). Histograms show that all three predictors have normal distribution of prediction errors. However, the normal distribution for LsRP shows a smaller standard deviation (S.D. _LsRP_ = 2.04 min) than SSRCalc (S.D. _SSRCalc_ = 6.05 min) and ELUDE (S.D. _ELUDE_ = 6.67 min). It is indicated that LsRP can predict RTs more accurately.

**Figure 3:**
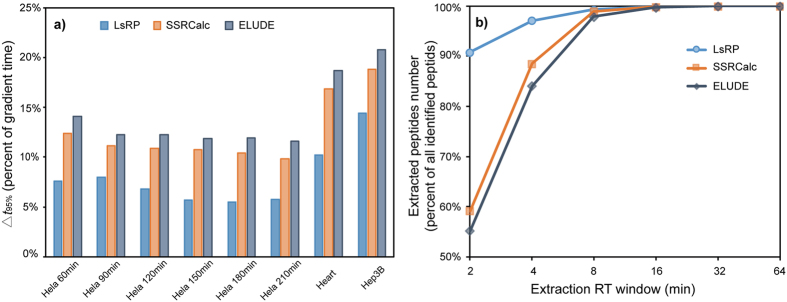
Prediction performances of LsRP, SSRCalc and ELUDE for 8 data sets. (**a**) Performances of LsRP, SSRCalc and ELUDE were evaluated in terms of the time window of the predicted time that would include the observed RTs of the peptide in 95% of the cases (Δ*t*_95%_) in different data sets. The percentage that Δ*t*_95%_ represents out of the total gradient time was displayed. (**b**) Capacities of LsRP, SSRCalc, and ELUDE. The diagram shows the extracted peptides percent distributions in different extraction RT windows for Hela 210 min data set.

To confirm the outstanding performance of LsRP, it was further applied on other 7 data sets of different chromatographic conditions or different real samples. After removing the peptides with more than 25 amino acids, each data set were divided into equally sized training and test sets in the same way as Hela 210 min data set. For all predictors (LsRP, SSRCalc and ELUDE), the training peptides were used to train a prediction model, which were then employed to predict RTs for the remaining test peptides. Pearson’s correlation *r* between predicted and observed RTs of the test peptides was used to evaluate the performance of three predictors. The minimal time windows of extraction including the deviations between predicted and observed RTs for 95% of the peptides (Δ*t*_95%_) were also introduced into the evaluations (Fig. 3a). LsRP was found to yield better correlations and narrower time windows for all data sets as well. Supplementary Table S2 shows that LsRP yielded better Pearson’s correlations between 0.95 and 0.99, compared with correlations between 0.91–0.96 by SSRCalc and ELUDE. In addition, LsRP had narrower cover windows corresponding to 5.5–14.4% of the total gradient time in different test sets, compared with 9.8–18.8% of SSRCalc and 11.6–20.8% of ELUDE. In these cases, when both SSRCalc and ELUDE showed similar prediction accuracies, LsRP provided smaller error windows and more accurate predictions.

Besides, when results were expressed in terms of peptide tracking capacity in either SRM or SWATH method, the difference between three predictors becomes clearer. A lower Δ*t*_95%_ window means that the MS instruments select smaller time intervals for targeted peptides, which implies that a larger number of peptides can be monitored in one MS run^40^,^41^. Figure 3b shows an increasing peptide tracking capacity achieved by LsRP compared with SSRCalc and ELUDE in the Hela 210 min experiment. Within an extraction window of 2 min, LsRP was able to track about 30% extra peptides than SSRCalc and ELUDE. The differences of capacity between LsRP and other predictors decreased as the time window get larger until the extraction window reaches 8 min.

Furthermore, peptides longer than 25 residues were introduced for testing. Although those peptides longer than 25 residues were not considered in the model at first, it is of interest whether LsRP can be applied to longer peptides. The corresponding vectors were translated only by first 13 and last 12 amino acids for these longer peptides, and then used for RT prediction with the pre-trained models. Supplementary Table S3 shows that LsRP also offered a high prediction accuracy for longer peptides. In each data set, there are at least 90% of longer peptides that have a deviation of predicted and observed RTs less than Δ*t*_95%_. In other words, the prediction accuracy of LsRP was barely affected by the ignored middle residues of longer peptides.

### Robustness test and Model Calibration

**Figure 4:**
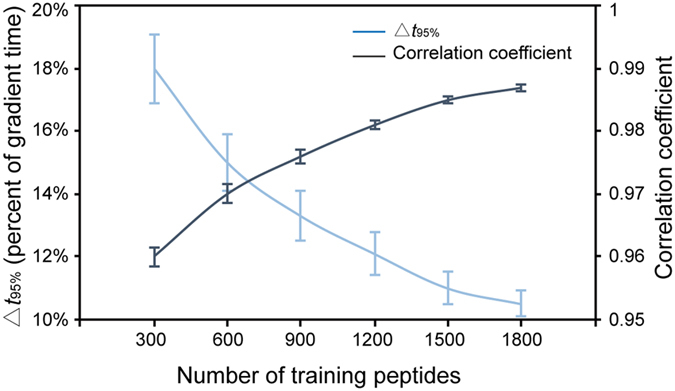
Prediction performances of LsRP with fewer training peptides for Heart data set. Performances of LsRP with different numbers of training peptides, in terms of the percentage that Δ*t*_95%_ represents out of the whole gradient time (blue) and correlations coefficient (black). Each point represents the average value of 100 runs, while the bars illustrate the standard deviation.

To demonstrate that LsRP is robust enough for predicting with small training sets, different sizes (from 300 to 1800 peptides) of random peptides from Heart data set were selected for training and remaining peptides for testing. This evaluation of each training size was repeated 100 times. The mean correlation coefficients and Δ*t*_95%_ of these 100 runs were plotted as well as the standard deviation (Fig. 4). Although, the result shows that prediction accuracy decreases with fewer peptides used for training, LsRP with 300 training peptides still offered a high correlation of 0.961 between observed and predicted RTs, as well as a Δ*t*_95%_ window of 18.1% of the total gradient time. It is showed that the prediction accuracy of LsRP is high enough for training with less data. For comparison, SSRCalc and ELUDE were also evaluated with the training size of 300 peptides. The Δ*t*_95%_ of ELUDE increased to 29.2% of the gradient running time, while the correlation coefficient decreased to 0.945. As for SSRCalc, the Δ*t*_95%_ increased to 26.3% and the correlation coefficient decreased to 0.949. It is found that LsRP still provided better prediction results than SSRCalc and ELUDE with small training sets. Meanwhile, it has to be mentioned that a well-trained SSRCalc model with nearly 1,000,000 training peptides has been available on the web server since 2015, which is considered to provide a prediction correlation coefficient higher than 0.97. This suggests that for machine learning process, more training peptides offered better prediction accuracy.

Although LsRP showed a good prediction accuracy with either large or small training sets, measurements from different laboratories are often under different conditions or with different machines, which requires calibrating the well-trained model to different samples. In addition, the targeted proteomics experiments usually prefer a workflow of running only a simple mixture of peptides prior to experiments. In this case, the identified peptides of first experiment are used to calibrate a specific model, which predicts the RTs of interested peptides for the second SRM or SWATH experiment.

**Figure 5:**
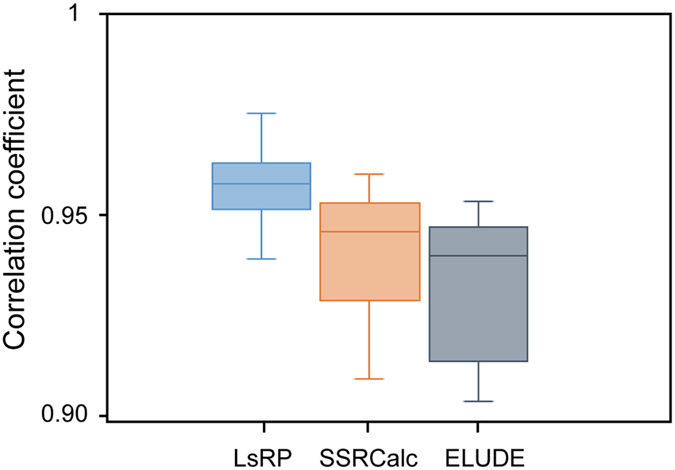
Prediction accuracies of pre-trained models applied to different LC conditions. The accuracy of the predictions when models and test sets were generated under different chromatographic conditions (different gradients) was calculated in terms of Pearson’s correlations for LsRP, SSRCalc and ELUDE.

Therefore, whether a pre-trained LsRP model can still accurately predict RTs under different LC conditions was firstly checked via inspecting the correlations. Considering the similarity of samples, 6 different Hela data sets were investigated. RTs of the test peptides were predicted using models built with training peptides from different data sets. A high correlation between observed and predicted RT (average *r* = 0.96) was obtained for all the combinations of training and test sets (Table S4). SSRCalc and ELUDE were also used as comparisons. Figure 5 shows that when predicting RTs under different LC conditions with a pre-trained model, LsRP yielded better Pearson’s correlations and a smaller standard deviation than other predictors (average *r* = 0.946 for SSRCalc and average *r* = 0.938 for ELUDE).

**Figure 6:**
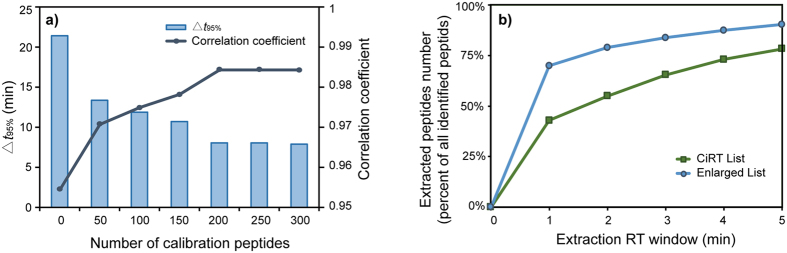
Performances of LsRP when calibrating a pre-trained model to Heart data set. (**a**) Performance of LsRP were evaluated in terms of Δ*t*_95%_ and correlations coefficient between predicted and observed RTs. Different numbers of peptides were employed to calibrate the model trained with Hela 210 min data set. (**b**) Capacities of different calibration lists. The diagram shows the extracted peptides percent distributions in different extraction RT windows.

To further evaluate the prediction accuracy for different samples, peptide RTs from Heart data set were predicted by a model trained from Hela 210 min data set. Different numbers of internal calibration peptides were employed (see Methods Part). The value of Δ*t*_95%_ were then utilized to demonstrate the prediction accuracy (Fig. 6a). The result shows that the Δ*t*_95%_ window will be relatively larger without any calibration, because two data sets have different gradient slopes. It is also showed that more calibration peptides will lead to smaller Δ*t*_95%_ windows, which indicates better predictions. Figure 6a suggests that although the LsRP with 50 calibration peptides showed a good prediction performance, using a set of at least 200 internal calibration peptides was sufficient enough to provide a high prediction accuracy in this method.

Recently, Parker presented a list of common peptides highly conserved among most eukaryotes along with their normalized RT values (termed CiRT, for common iRT peptides)^7^. These 113 peptides are reported to be present in a range of eukaryotes from yeast to human across a number of experimental preparations and thus can be used to convert raw peptide RTs into a normalized iRT values. There were half of these 113 peptides found in the Heart data set. To evaluate the feasibility of combing LsRP and enlarged calibration peptide list, 50 peptides from CiRT list and 200 internal peptides were separately picked out from the Heart data set and used to calibrate the pre-trained model (see Supplementary Tables S5 and S6). The RTs of remaining peptides of Heart data set were predicted and compared with the observed RTs (see Methods part). Figure 6b shows more than 80% peptides can be extracted in a 2 min RT window with the enlarged calibration list. This suggests that there is little loss in accuracy when employing a pre-trained LsRP model with a careful calibration.

In summary, this new predictor, LsRP combining locus vectors and SVR algorithm, has been successfully proposed for precision peptide RT prediction. Different from other predictors based on peptide sequence, this predictor takes advantage of the whole information of amino acid locus in terms of locus vectors. LsRP delivers excellent results for different data sets, outperforming common predictors like SSRCalc and ELUDE. Although a small training set of 300 peptides for LsRP can provide a reliable prediction accuracy, it is still suggested more training peptides are needed for a well-trained prediction model of LsRP. A good RT normalization process (e.g. iRT approach) might help LsRP employ a large training set consisting of different experiments in future.

LsRP can also be used in targeted proteomics or DIA assays, where small sets of training or calibration peptides are available prior to initializing the experiments. In addition, using LsRP appended with calibration peptides, RTs of peptides from different samples can be predicted accurately. LsRP is also proved to be appropriate for dealing with data under different gradients. The high prediction accuracies for different samples and LC conditions dramatically enlarged the applicable area of LsRP. It should be mentioned that LsRP might have the potential to help SWATH and DIA experiment design and open up new opportunities for precision proteomics.

In a chromatographic system, a large number of factors such as the three-dimensional structure of the peptide and detailed physicochemical properties influence the peptide elution. Peptide sequence information can be used to calculate a part of the properties with the locus vectors employed by LsRP, which provides accurate prediction results. However, more structural information that cannot be calculated simply with peptide sequence and locus vectors are required for predicting a real elution process. It is expectable that LsRP would yield even better predictions, combined with new methods which can apply peptide structural information in large scales.

All source code of LsRP and data sets have been deposited to the ProteomeXchange with identifier <PXD005572>.

## Methods

### Chemicals and Reagents

Formic acid, acetonitrile, penicillin, streptomycin, dithiothreitol (DTT) and iodoracetamide (IAA) were purchased from Sigma (St. Louis, MO, USA). Sequencing grade trypsin was purchased from Promega Corporation (Madison, USA). Pierce RT calibration mixture was purchased from Thermo Scientific (Waltham, MA, USA). Pure water was prepared with a Milli-Q system (Millipore, Bedford, MA, USA).

The human heart sample was kindly provided by Fudan University Shanghai Zhongshan hospital. Informed consent was gained from each participant. The research followed the tenets of the Declaration of Helsinki and was approved by the Ethics Committee of the Fudan University Shanghai Zhongshan hospital.

### Sample Preparation

Hela and Hep3B cells were cultured in Dulbecco’s Modified Eagle Medium (DMEM) supplemented with 10% fetal bovine serum (FBS) (Biowest, South America Origin), 100 U/mL penicillin, and 100 μg/mL streptomycin at 37 °C in 5% CO_2_. Cells were collected and dissolved in cell lysis buffer (8 M Urea, 2 M Thiourea, 65 mM DTT, 1 mM PMSF), and incubated on ice for 45 min with interval vortex. The lysate was centrifuged at 12000 g for 15 min at 4 °C, and the supernatants were harvested. Protein concentration was determined by Bradford assay. 100 μg protein of each cell line lysate were applied for further tryptic digestion at 37 °C overnight after reduction and alkylation.

### RPLC-MS/MS Conditions

Samples added with Pierce RT calibration mixture were analyzed on Triple TOF 5600 mass spectrometry (Applied Biosystems, USA) equipped with Eksigent nano-LC 425 system (Eksigent Technologies, USA). The instrument was controlled by Analyst TF 1.7 (Applied Biosystems, USA). The chromatographic separation of the peptides mixtures was achieved using a 50 cm RP analytical column (C18, 2 μm, 500 mm × 75 μm, Thermo Scientific, USA) at the flow rate of 200 nL/min and the linear gradient was from 0% to 30% of solvent B. The mobile phases: solvent A – 0.1% aqueous solution of formic acid, and solvent B – 0.1% formic acid in MS-grade acetonitrile were mixed on-line. Column oven temperature was set to 40 °C. The procedure was repeated 10 times of a 60, 90, 120, 150, 180, and 210 min gradient time for Hela sample, and a 150 min gradient time for both human heart sample and Hep3B sample.

Triple TOF 5600 mass spectrometry was operated in data-dependent mode to switch automatically between MS and MS/MS acquisition. Survey scans were acquired from 350–1250 in high resolution mode using 250 ms accumulation time per spectrum. Up to 20 precursors were selected for MS/MS scan from m/z 100–2000 in high sensitivity mode using a dynamic exclusion of 9 s.

### Protein Identification

Raw profile mode files in wiff format were transformed to mgf format. Peptide sequences were automatically searched against protein database (a fasta file downloaded from UniProtKB) appended with common contaminants and decoy sequences with Mascot (Matrix Science, UK). Tryptic peptides with up to two missed cleavages were allowed, with carbamidomethylation of cysteines set as a fixed modification and variable modifications of oxidation. Precursor and product ion mass errors were set to 50 ppm and 0.5 Da. Search engine results were converted to dat format. Peptide spectral match probability scoring and FDR value was then modeled in Scaffold 4 (Proteome Software, USA). Peptide identification probability was set as at least 90% and FDR was set as 1%. Observed RTs of the identified peptides were defined at peak intensity maximum.

### Amino Acid Locus Recognition

Features derived from the peptide’s sequence and amino acid composition were introduced into LsRP. Each amino acid residue is coded as a 20-dimensional binary vector consisting of 19 zero values and 1 one value that corresponds to the amino acid residue occupying that position. For each peptide, amino acid residues were positioned starting from two terminals to the center. Using a 7-residue peptide FEGIIYR as an example, the amino acid residues FEGI are positioned at the first 4 vectors, while the amino acid residues IYR are positioned at the last 3 vectors, leaving the center vectors filled with zero values. Peptides of a maximum length of 25 were chosen to produce information, filling all the 25 vectors that consists of 500 values.

### Support Vector Regression

The *ε* support vector regression (*ε*-SVR) functionality in the libsvm package was used in the RT predictor. A radial basis function kernel was employed and optimization of SVR parameters is crucial to avoid overfitted or underfitted models. The best appropriate values of the parameters *C, γ* and *ε* were figured out via 3-fold internal cross-validation, with *C* ∈ {2^*i*^|*i* ∈ {−8, −7, …, 8}}, *γ* ∈ {2^*i*^|*i* ∈ {−8, −7, …, 8}}, *ε* ∈ {10^*i*^|*i* ∈ {−3, −2, −1}}. For each data set, the training peptides were used to train the SVR, while the test peptides to evaluate prediction performance. Both amino acid locus recognition and SVR were operated in the Matlab R2016a (The MathWorks, Inc., United States).

### Retention Time Calibration

To apply a pre-trained model with Hela 210 min data set to Heart data set, the following calibration steps were performed. First, a series of internal peptides widely identified from online heart spectral libraries (PeptideAtlas) were selected as the calibration peptides in the descending order of their abundance and identification times. Based on the spectral libraries online, observed RTs of these calibration peptides can be extracted and confirmed from Heart data set. Second, RTs of these calibration peptides were predicted with the pre-trained model and put into a linear regression with their observed RTs in Heart data set. Different amounts of calibration peptides were employed to calculate the value of A and B of the regression “Observed RT = A × Predicted RT + B”. Then, RTs of all identified peptides from Heart data set were predicted and converted to calibrated RTs using a linear regression “Calibrated RT = A × Predicted RT + B”. Calibrated RTs were finally used to compared with observed RTs for all identified peptides.

### Benchmarked Methods

Two commonly used predictors were used to benchmark with our new RT predictor LsRP in this work. SSRCalc: the latest SSRCalc version available was employed at the website http://hs2.proteome.ca/SSRCalc/SSRCalcQ.html. With the trained model of “100 Å C18 column, 0.1% Formic Acid 2015”, the HI value of each peptide were presented after submitting the peptide sequences. The SSRCalc requires two user calculated regression coefficients *a* and *b*, that formed a linear function between RTs and HI values. We employed the predicted HI values of the training peptides together with the observed RTs to derive the coefficients *a* and *b* via the least-squares regression. Then the resulting coefficients were utilized to predict RTs for the test peptides.

ELUDE: the ELUDE was applied via the web server at http://elude.sbc.su.se. Test data were pasted into the first blank table while training data into the second table with the selection of “Train a model”. After submitting the query, predicted RTs for test data can be downloaded.

## Additional Information

**How to cite this article:** Lu, W. *et al*. Locus-specific Retention Predictor (LsRP): A Peptide Retention Time Predictor Developed for Precision Proteomics. *Sci. Rep.* **7**, 43959; doi: 10.1038/srep43959 (2017).

**Publisher's note:** Springer Nature remains neutral with regard to jurisdictional claims in published maps and institutional affiliations.

## Supplementary Material

Supplementary Information

## Figures and Tables

**Table 1:** Data sets under different chromatographic conditions to evaluate the performance of LsRP.

Data set	Gradient time	Peptides	Peptides (length ≤ 25)	Training peptides	Test peptides
Hela 60 min	60	5630	5446 (96.7%)	2723	2723
Hela 90 min	90	6236	6089 (97.6%)	3044	3045
Hela 120 mn	120	8535	8273 (96.9%)	4137	4136
Hela 150 min	150	8694	8560 (98.4%)	4280	4280
Hela 180 min	180	11688	11361 (97.2%)	5681	5680
Hela 210 min	210	11523	11237 (97.5%)	5619	5618
Heart	150	4118	3984 (96.7%)	1992	1992
Hep3B	150	3768	3670 (97.4%)	1845	1845
