# Supplementary material for: Locus-specific Retention Predictor (LsRP): A Peptide Retention Time Predictor Developed for Precision Proteomics
Source: Sci Rep. 2017 Mar 17;7:43959. doi: 10.1038/srep43959 (PMC5356008; doi:10.1038/srep43959)
Supplement: Supplementary Information [file srep43959-s1.pdf]

# Supporting Information

## Locus-specific Retention Predictor (LsRP): A Peptide Retention Time Predictor Developed for Precision Proteomics

**Wenyuan Lu[1], Xiaohui Liu[2], Shanshan Liu[1], Weiqian Cao[1], Yang Zhang[1] & Pengyuan Yang[1, 2]**

[1] Institutes of Biomedical Sciences and Department of Systems Biology for Medicine, School of Basic Medical, Fudan University, Shanghai 200032, P. R. China.

[2] Department of Chemistry, Fudan University, Shanghai 200433, P. R. China.

Correspondence and requests for materials should be addressed to P.Y. (email: pyyang@fudan.edu.cn)

**Table S1.** Vectors corresponding to each amino acid.

| Amino Acid | Binary vector corresponding to each amino acid | | | | | | | | | | | | | | | | | | | |
|---|---|---|---|---|---|---|---|---|---|---|---|---|---|---|---|---|---|---|---|---|
| A | 1 | 0 | 0 | 0 | 0 | 0 | 0 | 0 | 0 | 0 | 0 | 0 | 0 | 0 | 0 | 0 | 0 | 0 | 0 | 0 |
| C | 0 | 1 | 0 | 0 | 0 | 0 | 0 | 0 | 0 | 0 | 0 | 0 | 0 | 0 | 0 | 0 | 0 | 0 | 0 | 0 |
| D | 0 | 0 | 1 | 0 | 0 | 0 | 0 | 0 | 0 | 0 | 0 | 0 | 0 | 0 | 0 | 0 | 0 | 0 | 0 | 0 |
| E | 0 | 0 | 0 | 1 | 0 | 0 | 0 | 0 | 0 | 0 | 0 | 0 | 0 | 0 | 0 | 0 | 0 | 0 | 0 | 0 |
| F | 0 | 0 | 0 | 0 | 1 | 0 | 0 | 0 | 0 | 0 | 0 | 0 | 0 | 0 | 0 | 0 | 0 | 0 | 0 | 0 |
| G | 0 | 0 | 0 | 0 | 0 | 1 | 0 | 0 | 0 | 0 | 0 | 0 | 0 | 0 | 0 | 0 | 0 | 0 | 0 | 0 |
| H | 0 | 0 | 0 | 0 | 0 | 0 | 1 | 0 | 0 | 0 | 0 | 0 | 0 | 0 | 0 | 0 | 0 | 0 | 0 | 0 |
| I | 0 | 0 | 0 | 0 | 0 | 0 | 0 | 1 | 0 | 0 | 0 | 0 | 0 | 0 | 0 | 0 | 0 | 0 | 0 | 0 |
| K | 0 | 0 | 0 | 0 | 0 | 0 | 0 | 0 | 1 | 0 | 0 | 0 | 0 | 0 | 0 | 0 | 0 | 0 | 0 | 0 |
| L | 0 | 0 | 0 | 0 | 0 | 0 | 0 | 0 | 0 | 1 | 0 | 0 | 0 | 0 | 0 | 0 | 0 | 0 | 0 | 0 |
| M | 0 | 0 | 0 | 0 | 0 | 0 | 0 | 0 | 0 | 0 | 1 | 0 | 0 | 0 | 0 | 0 | 0 | 0 | 0 | 0 |
| N | 0 | 0 | 0 | 0 | 0 | 0 | 0 | 0 | 0 | 0 | 0 | 1 | 0 | 0 | 0 | 0 | 0 | 0 | 0 | 0 |
| P | 0 | 0 | 0 | 0 | 0 | 0 | 0 | 0 | 0 | 0 | 0 | 0 | 1 | 0 | 0 | 0 | 0 | 0 | 0 | 0 |
| Q | 0 | 0 | 0 | 0 | 0 | 0 | 0 | 0 | 0 | 0 | 0 | 0 | 0 | 1 | 0 | 0 | 0 | 0 | 0 | 0 |
| R | 0 | 0 | 0 | 0 | 0 | 0 | 0 | 0 | 0 | 0 | 0 | 0 | 0 | 0 | 1 | 0 | 0 | 0 | 0 | 0 |
| S | 0 | 0 | 0 | 0 | 0 | 0 | 0 | 0 | 0 | 0 | 0 | 0 | 0 | 0 | 0 | 1 | 0 | 0 | 0 | 0 |
| T | 0 | 0 | 0 | 0 | 0 | 0 | 0 | 0 | 0 | 0 | 0 | 0 | 0 | 0 | 0 | 0 | 1 | 0 | 0 | 0 |
| V | 0 | 0 | 0 | 0 | 0 | 0 | 0 | 0 | 0 | 0 | 0 | 0 | 0 | 0 | 0 | 0 | 0 | 1 | 0 | 0 |
| W | 0 | 0 | 0 | 0 | 0 | 0 | 0 | 0 | 0 | 0 | 0 | 0 | 0 | 0 | 0 | 0 | 0 | 0 | 1 | 0 |
| Y | 0 | 0 | 0 | 0 | 0 | 0 | 0 | 0 | 0 | 0 | 0 | 0 | 0 | 0 | 0 | 0 | 0 | 0 | 0 | 1 |

**Figure S1. Correlation between predicted and observed RTs for Hela 210min data set.** a) SSRCalc, b) ELUDE

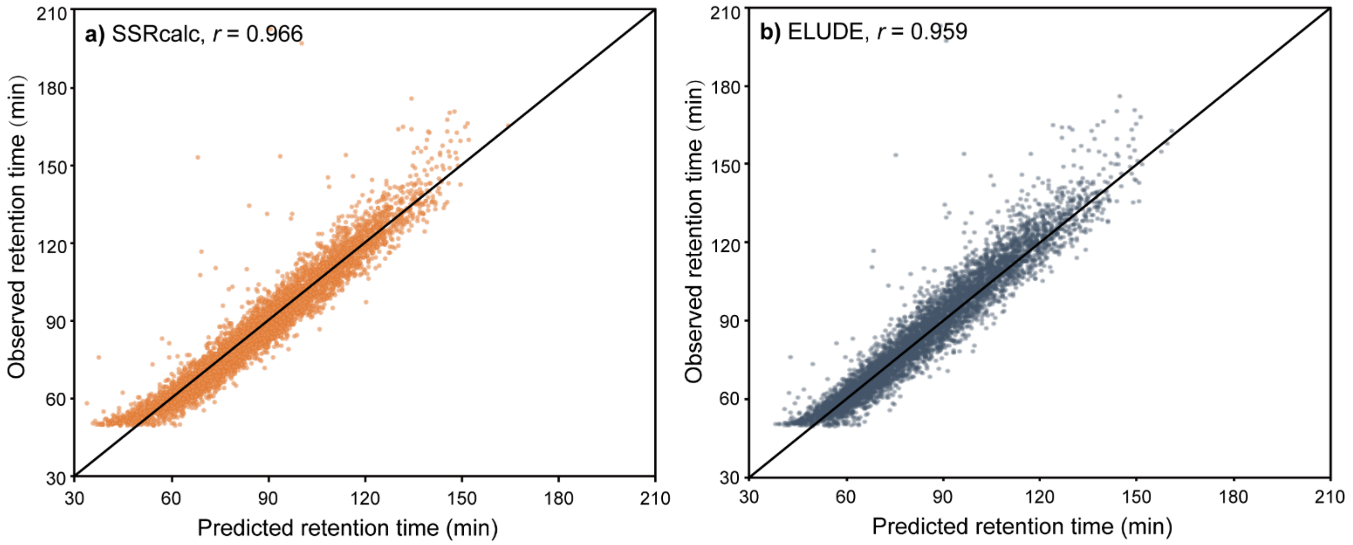


**Figure S2. Distribution of prediction error for Hela 210min data set.** a) SSRCalc, b) ELUDE

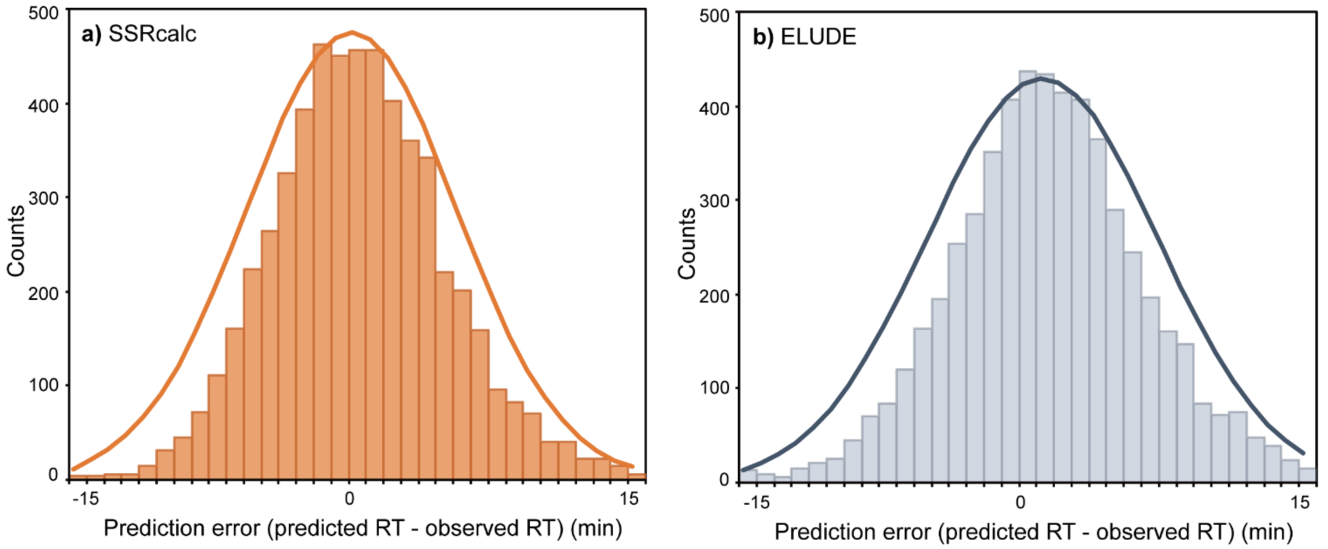

**Figure S3. Comparison of prediction errors of a) SSRCalc and b) ELUDE with LsRP.**

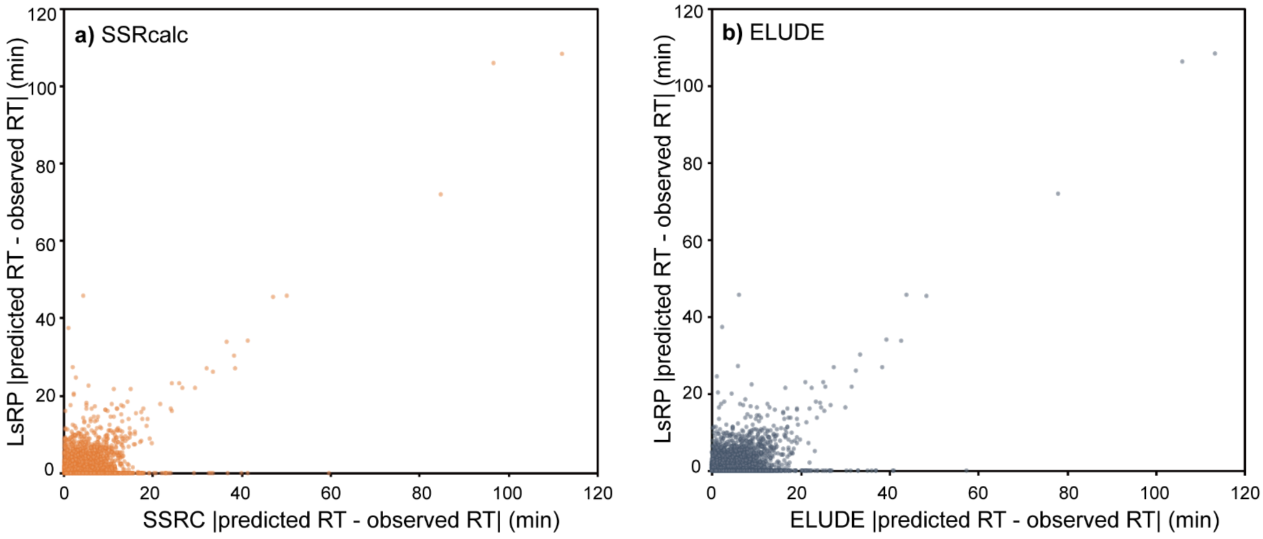


**Table S2.** Comparison between the performances of LsRP, SSRCalc, and ELUDE[a].

| Data set | $\Delta t_{95\%}$ | | | Pearson's correlation coefficient *r* | | |
|---|---|---|---|---|---|---|
| | SSRCalc | ELUDE | LsRP | SSRCalc | ELUDE | LsRP |
| Hela 60min | 7.4 min (12.4%) | 8.5 min (14.1%) | 4.6 min (7.6%)) | 0.913 | 0.907 | 0.959 |
| Hela 90min | 10.0 min (11.1%) | 11.1 min (12.3%) | 7.2 min (8.0%) | 0.947 | 0.941 | 0.973 |
| Hela 120mn | 13.0 min (10.8%) | 14.7 min (12.3%) | 8.1 min (6.8%) | 0.940 | 0.921 | 0.970 |
| Hela 150min | 16.2 min (10.8%) | 17.8 min (11.9%) | 8.5 min (5.7%) | 0.955 | 0.950 | 0.981 |
| Hela 180min | 18.7 min (10.4%) | 21.5 min (11.9%) | 9.9 min (5.5%) | 0.956 | 0.949 | 0.982 |
| Hela 210min | 20.6 min (9.8%) | 24.4 min (11.6%) | 12.1 min (5.8%) | 0.966 | 0.959 | 0.987 |
| Heart | 25.2 min (16.8%) | 28.0 min (18.6%) | 15.3 min (10.2%) | 0.969 | 0.965 | 0.989 |
| Hep3B | 28.2 min (18.8%) | 31.2 min (20.8%) | 21.6 min (14.4%) | 0.948 | 0.948 | 0.976 |

a) The performances of SSRCalc, ELUDE and LsRP were evaluated in terms of the time window of the predicted time that would include the observed retention times of the peptide in 95% of the cases ($\Delta t_{95\%}$). And the percentage that $\Delta t_{95\%}$ represents out of the whole gradient time was also displayed. In addition, the Pearson's correlation coefficients of three predictors were listed in the last three columns.

**Table S3.** Performances of peptides (length > 25) retention prediction.

| Data set | Number of Peptides (length>25) | Number of Peptides (length>25) in $\Delta t_{95\%}$ |
|---|---|---|
| Hela 60min | 184 | 176 |
| Hela 90min | 147 | 141 |
| Hela 120mn | 262 | 250 |
| Hela 150min | 134 | 129 |
| Hela 180min | 327 | 310 |
| Hela 210min | 286 | 278 |

| | | |
|---|---|---|
| Heart | 134 | 129 |
| Hep3B | 98 | 93 |

**Table S4.** Prediction accuracies of LsRP pre-trained model applied to different LC conditions.

The accuracy of the predictions when models and test sets were generated under different chromatographic conditions (different gradients) was calculated. The rows correspond to the training datasets used to generate a model, while the columns to the test ones. Pearson's correlations between predicted and observed retention times for the test peptides are displayed.

| | Hela 60min | Hela 90min | Hela 120min | Hela 150min | Hela 180min | Hela 210min |
|---|---|---|---|---|---|---|
| Hela 60min | 0.959 | 0.957 | 0.948 | 0.957 | 0.954 | 0.961 |
| Hela 90min | 0.964 | 0.973 | 0.949 | 0.961 | 0.957 | 0.963 |
| Hela 120min | 0.967 | 0.963 | 0.970 | 0.968 | 0.966 | 0.972 |
| Hela 150min | 0.966 | 0.964 | 0.955 | 0.981 | 0.967 | 0.974 |
| Hela 180min | 0.959 | 0.950 | 0.944 | 0.959 | 0.982 | 0.968 |
| Hela 210min | 0.965 | 0.962 | 0.955 | 0.969 | 0.970 | 0.987 |

**Table S5.** List of 50 CiRT peptides found in Heart data set.

| Peptide sequence | iRT value |
|---|---|
| EGIPPDQQR | -15.8411 |
| FDDGAGGDNEVQR | -11.317 |
| AGFAGDDAPR | -9.81926 |
| NMSVIAHVDHGK | -5.36295 |
| EIAQDFK | -4.04913 |
| ATAGDTHLGGEDFDNR | 3.185527 |
| TLSDYNIQK | 4.35 |
| VVPGYGHAVLR | 8.61752 |
| KPLLESGTLGTK | 9.057185 |
| QTVAVGVIK | 9.9 |
| LVLVGDGGTGK | 12.0229 |
| TTIFSPEGR | 15.18379 |
| VAVVAGYGDVGK | 15.3314 |
| AQIWDTAGQER | 16.85488 |
| VPAINVNDSVTK | 17.70942 |
| AVANQTSATFLR | 19.24765 |
| IGGIGTVPVGR | 21.9 |
| AFLIEEQK | 22.8 |
| ELISNASDALDK | 23.50069 |
| NLQYYDISAK | 25.8 |
| YLVLDEADR | 27.6947 |
| GVLLYGPPGTGK | 28.11667 |

| | |
|---|---|
| ESTLHLVLR | 28.54494 |
| LPLQDVYK | 29.2 |
| IGPLGLSPK | 29.48313 |
| TIAMDGTEGLVR | 32.82289 |
| AVFPSIVGRPR | 34.03497 |
| NLLSVAYK | 34.34229 |
| LQIWDTAGQER | 36.28872 |
| AGLQFPVGR | 37.04898 |
| FTVDLPK | 37.86026 |
| FDLMYAK | 38.2 |
| FVIGGPQGDAGLTGR | 40.55198 |
| IGLFGGAGVGK | 43.28519 |
| GILLYGPPGTGK | 45.36582 |
| LYQVEYAFK | 46.26582 |
| EAYPGDVFYLHSR | 46.35 |
| EMVELPLR | 47.96546 |
| NILGGTVFR | 49.61455 |
| GILFVGSGVSGGEEGAR | 51.15 |
| YPIEHGIVTNWDDMEK | 56.9 |
| DAGTIAGLNVLR | 59.03745 |
| DLTDYLMK | 60.01111 |
| FTQAGSEVSALLGR | 61.45034 |
| IINEPTAAAIAYGLDK | 65.72007 |
| SYELPDGQVITIGNER | 67.30002 |
| DWNVDLIPK | 70.53546 |
| VLPSIVNEVLK | 83.75009 |
| YFPTQALNFAFK | 95.4 |
| DSTLIMQLLR | 103.65 |

**Table S6.** List of 200 internal calibration peptides from Heart data set.

| Peptide sequence | Extracted RT (min)[a] | Calibrated RT (min) |
|---|---|---|
| AAAEVNQDYGLDPK | 82.6742 | 83.32082 |
| AAAVLPVLDLAQR | 125.7367 | 126.0115 |
| AAFDDAIAELDTLSEESYK | 154.2776 | 153.269 |
| AAIISAEGDSK | 69.0104 | 68.71493 |
| AAMDNSEIAGEK | 68.5155 | 63.60409 |
| AAVDAGFVPNDMQVGQTGK | 104.5282 | 105.0647 |
| AAVPSGASTGIYEALELR | 123.9526 | 126.4831 |
| AAYEAELGDAR | 77.401 | 77.87696 |
| AAYFGVYDTAK | 94.5405 | 95.19862 |
| ADDGRPFPQVIK | 83.83503 | 84.63293 |
| ADIDVSGPK | 70.5101 | 70.43419 |

| | | |
|---|---|---|
| ADLIAYLK | 110.3807 | 110.586 |
| ADLTEYLSTHYK | 100.0993 | 100.1905 |
| ADNFEYSDPVDGSISR | 101.0921 | 101.8387 |
| ADSAVSQEQLR | 67.9685 | 67.23326 |
| ADVVESWIGEK | 106.8073 | 107.6855 |
| AEAGDNLGALVR | 91.54057 | 91.97203 |
| AEDGSVIDYELIDQDAR | 118.414 | 118.5843 |
| AEGDVAALNR | 70.0547 | 69.82407 |
| AEGPDVAVDLPK | 94.6257 | 95.54172 |
| AEGPEVDVNLPK | 93.1243 | 93.43363 |
| AELIVQPELK | 97.4234 | 98.09587 |
| AFAAGADIK | 72.72675 | 73.09904 |
| AFGPGLQGGSAGSPAR | 79.1372 | 79.98892 |
| AGFAGDDAPR | 69.4568 | 68.09798 |
| AGLGSGLSLSGLVHPELSR | 119.3889 | 119.3319 |
| AGLQFPVGR | 94.47503 | 95.57268 |
| AGLVDDFEK | 91.6264 | 92.77809 |
| AGQSAAGAAPGGGVDTR | 65.4548 | 64.46138 |
| AGTQIENIEEDFR | 107.5772 | 107.9825 |
| AGTQIENIEEDFRDGLK | 110.7399 | 116.1921 |
| AGVAAPATQVAQVTLQSVQR | 112.0575 | 112.5093 |
| AHGGYSVFAGVGER | 82.19023 | 83.02681 |
| AHSSMVGVNLPQK | 73.7446 | 68.41705 |
| AIAELGIYPAVDPLDSTSR | 136.314 | 135.2668 |
| AIAVLTSGGDAQGMNAAVR | 98.9087 | 99.29524 |
| AIEMLGGELGSK | 100.1121 | 100.652 |
| AILVDLEPGTMDSVR | 120.7755 | 114.7643 |
| AIPDLTAPVAAVQAAVSNLVR | 175.4443 | 176.8114 |
| AIVAGDQNVEYK | 76.34285 | 76.72312 |
| AIVAIENPADVSVISSR | 116.7344 | 117.0904 |
| ALAAAGYDVEK | 77.3161 | 81.4768 |
| ALDLFSDNAPPPELLEIINEDIAK | 176.2508 | 176.4997 |
| ALEQFATVVEAK | 104.2057 | 104.8316 |
| ALESPERPFLAILGGAK | 122.0678 | 122.2842 |
| ALGQNPTNAEVLK | 79.8935 | 80.84305 |
| ALGTEVIQLFPEK | 126.8349 | 127.0065 |
| ALIEVLQPLIAEHQAR | 123.7339 | 133.2228 |
| ALINADELASDVAGAEALLDR | 163.5932 | 167.9177 |
| ALLVEPVINSYLLAER | 144.0963 | 143.2337 |
| ALMLQGVDLLADAVAVTMGPK | 176.7536 | 184.6561 |
| ALPFWNEEIVPQIK | 141.2003 | 140.5054 |
| ALSEIAGMTLPYDTLDQVR | 139.8312 | 138.489 |

| | | |
|---|---|---|
| ALTSELANAR | 76.4344 | 77.01041 |
| ALTVPELTQQMFDAK | 131.974 | 131.6962 |
| ALTVPELTQQVFDAK | 129.8945 | 130.2471 |
| AMGIMNSFVNDIFER | 151.5456 | 148.5355 |
| ANLQIDQINTDLNLER | 119.3144 | 119.0049 |
| ANTFVAELK | 86.8493 | 87.92745 |
| APDFVFYAPR | 113.9779 | 114.6401 |
| APDVDVNIAGPDAALK | 105.6422 | 106.3623 |
| APIIAVTR | 78.9837 | 79.88158 |
| APIQWEER | 80.636 | 81.34838 |
| APLDIPVPDPVK | 116.107 | 116.7502 |
| APMVNPTLGVHEADLLK | 106.1508 | 106.6735 |
| APPSVFAEVPQAQPVLVFK | 138.219 | 136.9166 |
| APSVPAAEPEYPK | 81.6558 | 82.39255 |
| APVPASELLASGVLSR | 120.9264 | 121.2006 |
| AQAELVGTADEATR | 79.96795 | 78.01547 |
| AQDEGLLSDVVPFK | 131.2236 | 130.9355 |
| AQFAQPEILIGTIPGAGGTQR | 128.9964 | 128.5168 |
| AQFEGIVTDLIR | 130.7213 | 130.3296 |
| AQHEDQVEQYK | 63.5942 | 62.03056 |
| AQIHDLVLVGGSTR | 88.2254 | 89.00033 |
| AQPVQVAEGSEPDGFWEALGGK | 132.1649 | 131.1056 |
| AQQATPGGAAPTIFSR | 91.4587 | 92.41422 |
| AQQVSQGLDVLTAK | 95.5102 | 95.98126 |
| AQYPIADLVK | 103.6226 | 104.521 |
| ASAAPKPEPVPVQK | 65.86275 | 64.91701 |
| ASAELALGENSEVLK | 100.5983 | 100.3224 |
| ASAFALQEQPVVNAVIDDTTK | 132.5896 | 131.6565 |
| ASGPPVSELITK | 91.8349 | 93.06518 |
| ASNTAEVFFDGVR | 107.4898 | 108.2744 |
| ASSIIDELFQDR | 137.4557 | 136.5876 |
| ASSTSPVEISEWLDQK | 123.6344 | 123.5358 |
| ASWSSLSMDEK | 93.0377 | 93.02648 |
| ATAAPAGAPPQPQDLEFTK | 99.16668 | 99.42845 |
| ATAVVDGAFK | 77.6463 | 78.61703 |
| ATDAEADVASLNR | 80.99577 | 80.84784 |
| ATEPVIAFYEK | 102.796 | 103.5424 |
| AVAFQNPQTHVIENLHAAAYR | 95.07045 | 95.2142 |
| AVAGNISDPGLQK | 76.44145 | 76.98169 |
| AVDEAADALLK | 93.99457 | 94.93649 |
| AVDSLVPIGR | 97.55931 | 98.1143 |
| AVFPSIVGR | 100.8052 | 101.9424 |

| | | |
|---|---|---|
| AVFPSIVGRPR | 86.96201 | 87.28688 |
| AVFVDLEPTVIDEIR | 140.6947 | 139.7172 |
| AVFVDLEPTVIDEVR | 134.5678 | 134.1811 |
| AVLLGPPGAGK | 83.2808 | 84.27506 |
| AVLVDLEPGTMDSVR | 115.7231 | 109.4893 |
| AVTEQGAELSNEER | 70.0705 | 70.3961 |
| AVTEQGHELSNEER | 64.5029 | 62.97298 |
| AVTPAPPIK | 72.6422 | 73.27563 |
| AYGENIGYSEK | 73.7935 | 74.18852 |
| AYPDVAALSDGYWVVSNR | 136.2977 | 135.2657 |
| DAGIEPGPDTYLALLNAYAEK | 161.9135 | 159.9864 |
| DAGMQLQGYR | 84.2526 | 84.61816 |
| DAGQISGLNVLR | 108.5285 | 109.0228 |
| DAGTIAGLNVLR | 115.8757 | 116.3602 |
| DAGTIAGLNVMR | 108.0244 | 108.6371 |
| DAGVIAGLNVLR | 124.1794 | 124.158 |
| DAINQGMDEELERDEK | 86.9578 | 87.18096 |
| DALDGPAAEAEPEHSFDGLR | 103.2138 | 103.4973 |
| DALNIETAIK | 103.3442 | 104.1987 |
| DASVAEAWLLGQEPYLSSR | 150.783 | 149.1588 |
| DATNVGDEGGFAPNILENK | 118.0361 | 119.009 |
| DDGSWEVIEGYR | 119.4289 | 119.342 |
| DDTIYEDEDVK | 84.2626 | 84.18096 |
| DFPLSGYVELR | 134.6707 | 133.345 |
| DFSPSGIFGAFQR | 143.817 | 142.92 |
| DFSSVFQFLR | 159.1394 | 157.5656 |
| DFTATDLSEFAAK | 122.7772 | 122.5306 |
| DGDSVMVLPTIPEEEAK | 127.9023 | 127.3894 |
| DGEQHEDLNEVAK | 67.7632 | 67.02958 |
| DGVVEITGK | 79.11605 | 79.60325 |
| DILADLIPK | 141.5243 | 140.2274 |
| DINQEVYNFLATAGAK | 155.416 | 153.1122 |
| DIVPGDIVEIAVGDK | 145.1227 | 143.5789 |
| DLADELALVDVIEDK | 163.0724 | 161.8806 |
| DLASVQALLR | 125.0513 | 124.8872 |
| DLDDFQSWLSR | 143.7145 | 143.5474 |
| DLEALLNSK | 114.1489 | 114.5484 |
| DLEALMFDR | 132.0678 | 132.0456 |
| DLFDPIIEDR | 132.4396 | 132.3928 |
| DLIGVQNLLK | 128.6541 | 128.4659 |
| DLLDDLK | 114.6077 | 114.9354 |
| DLLLDPAWEK | 127.1232 | 127.1287 |

| | | |
|---|---|---|
| DLNSDMDSILASLK | 161.5393 | 159.4914 |
| DLPVTEAVFSALVTGHAR | 145.5272 | 144.8499 |
| DLQANVEHLVQK | 97.42845 | 97.82742 |
| DLSLDDFK | 112.1255 | 112.5659 |
| DLTDYLMK | 121.7349 | 111.124 |
| DLVPDLSNFYAQYK | 143.381 | 141.9352 |
| DLYANTVLSGGTTMYPGIADR | 134.6095 | 137.8963 |
| DNHLLGTFDLTGIPPAPR | 130.7141 | 129.8132 |
| DPDAQPGGELMLGGTDSK | 102.6811 | 103.2182 |
| DPDMVQNTVSELIK | 133.2876 | 132.3857 |
| DPEAPIFQVADYGIVADLFK | 177.0484 | 184.2223 |
| DPENFPFVVLGNK | 137.3718 | 136.8544 |
| DPSASPGDAGEQAIR | 74.8456 | 75.24644 |
| DPVQEAWAEDVDLR | 124.0653 | 124.4035 |
| DPWYSWDQPGLR | 128.1003 | 127.2896 |
| DQLLLGPTYATPK | 109.6684 | 109.9983 |
| DQTDDQVTIDSALATQK | 103.9338 | 103.7753 |
| DSIFSNLTGQLDYQGFEK | 147.5196 | 145.8775 |
| DSLSDDVVK | 79.3697 | 79.92731 |
| DSTLIMQLLR | 144.5798 | 140.6174 |
| DSYVGDEAQSK | 67.81214 | 66.55113 |
| DTHDQLSEPSEVR | 69.9832 | 69.81134 |
| DTPGFIVNR | 91.5674 | 92.1369 |
| DTSASAVAVGLK | 85.6264 | 86.21929 |
| DVDEIEAWISEK | 138.7168 | 137.1131 |
| DVEDEILWVGER | 135.3613 | 134.5111 |
| DVTGAEALLER | 106.3016 | 106.9746 |
| DVVTAAGDMLK | 108.9055 | 109.86 |
| DYGVLLEGSGLALR | 136.9365 | 136.0255 |
| DYPVVSIEDPFDQDDWGAWQK | 161.6312 | 160.8408 |
| EAAENSLVAYK | 78.7484 | 79.67872 |
| EAALGAGFSDK | 79.5428 | 80.78969 |
| EAFQPQEPDFPPPPPDLEQLR | 133.5589 | 132.4682 |
| EAGAGGLSIAVEGPSK | 94.92365 | 94.23583 |
| EALTYDGALLGDR | 107.0266 | 107.7817 |
| EANLAASFGK | 82.6162 | 83.06123 |
| EAVAPVQEESDLEK | 82.1302 | 82.91438 |
| EAYMGNVLQGGEGQAPTR | 94.76883 | 95.07611 |
| EAYPGDVFYLHSR | 99.67288 | 100.1646 |
| EDLQELNDR | 75.5976 | 76.45823 |
| EDLRLPEGDLGK | 89.71055 | 90.30032 |
| EDPNLVPSISNK | 92.75538 | 93.17455 |

| | | |
|---|---|---|
| EEAENTLQSFR | 85.10715 | 86.00369 |
| EEGVLTLWR | 118.0729 | 118.7707 |
| EEIIPVAAEYDK | 102.6412 | 103.4955 |
| EFADSLGIPFLETSAK | 147.0553 | 146.5191 |
| EFDELNPSAQR | 86.44385 | 87.12363 |
| EFPGFLENQK | 104.7057 | 105.5149 |
| EFQDAGEQVVSSPADVAEK | 96.30965 | 96.85424 |
| EGDLIAAQAR | 74.4086 | 74.99326 |
| EGGLGPLNIPLLADVTR | 155.7149 | 154.3271 |
| EGMAALQSDPWQQELYR | 123.2407 | 123.0266 |
| EGNDLYHEMIESGVINLK | 130.0144 | 128.9924 |
| EHALLAYTLGVK | 98.757 | 99.57285 |
| EIDDSVLGQTGPYR | 101.0854 | 101.745 |
| EIDGGLETLR | 91.6515 | 92.22988 |
| EIEQEAAVELSQLR | 115.63 | 115.7939 |
| EIGQSVDEVEK | 72.9932 | 73.24884 |
| EILVGDVGQTVDDPYATFVK | 134.533 | 134.062 |
| EIRPALELLEPIEQK | 118.5116 | 118.8359 |
| EITALAPSTMK | 88.21162 | 82.94157 |
| EIVDSYLPVILDIIK | 176.0878 | 176.4997 |
| EIVTNFLAGFEA | 166.7943 | 162.8654 |

a) Peptide retention times were extracted based on M/z and spectra library online.
